# Reply: Increased risk of breast cancer among female relatives of patients with Ataxia-Telangiectasia: a causal relationship?

**DOI:** 10.1038/sj.bjc.6602785

**Published:** 2005-09-13

**Authors:** J H Olsen, J M D Hahnemann, A-L Børresen-Dale, S Tretli, R Kleinerman, R Sankila, L Hammarström, T E Robsahm, H Kääriäinen, A Bregård, K Brøndum-Nielsen, J Yuen, M Tucker

**Affiliations:** 1 Danish Cancer Society, Institute of Cancer Epidemiology, Strandboulevarden 49, Copenhagen DK-2100, Denmark

**Sir**,

Thank you for the opportunity to comment on this interesting letter, which addresses some important methodological issues in studies of risk factors with *post hoc* genotyping. In their collection of French families in which one or more child is affected by AT, d'Almeida and co-workers have shown how potential biases, introduced by late genotyping of relatives with certain outcomes, can be addressed by various analytical approaches. They then compare and discuss the results.

In the Nordic study, genotyping of probands and parents was generally completed during the diagnostic work-up of the AT patients, that is, at the date of start of follow-up for subsequent breast and other cancers. Supplementary genotyping of other family members was usually conducted years or decades later, either among survivors who were willing to participate or among relatives who had died from breast cancer and for whom tissue blocks were available. As the study hypothesis was that carriers of an *ATM* allele are at increased risk for breast cancer and perhaps other potentially deadly diseases, we considered that we could not backdate the result of the gene testing, that is, reallocate the person-years at risk from the start of follow-up of these relatives, without running the risk of introducing differential misclassification. As the date of testing was not available for all relatives, we decided not to change the gene probability scores of the tested persons but only to change the scores of their ancestors. We thus chose to retain some random gene exposure misclassification due to the initial allocation of carrier probability, defined by location in a family, rather than risk introducing non-random misclassification, which can lead to overestimation of risks.

It is reassuring that d'Almeira and co-workers report in their letter that some, limited variation in breast cancer risk estimates was found with each of the three approaches in the French material, and that the mothers in this study – as in the Nordic study – clearly showed a very high risk for breast cancer. In the Nordic study, we concluded that our data did not convincingly point to a trend of increasing risk with each increment in the probability of being a gene carrier, indicating that we should consider other mechanisms than a genetic one as the cause of breast cancer in these families. We nevertheless reported a significantly increased risk for breast cancer among female relatives below the age of 55 years who had an estimated gene carrier probability of 0.25, and we acknowledged that the estimated trend in breast cancer risk by increasing gene carrier probability was based on a very limited number of outcomes. As pointed out by d'Almeira and co-workers, international collaboration is the only means of addressing this problem in an epidemiological design.

